# Skin needling as a treatment for acne scarring: An up-to-date review of the literature^☆^

**DOI:** 10.1016/j.ijwd.2015.03.004

**Published:** 2015-04-10

**Authors:** Adam G. Harris, Catherine Naidoo, Dedee F. Murrell

**Affiliations:** Department of Dermatology, St George Hospital, Sydney, Australia; University of New South Wales, Sydney, Australia

**Keywords:** Acne scarring, Dermaroller, Microneedling, Micro-needling, Percutaneous collagen induction, Skin needling

## Abstract

**Background:**

Skin needling is a technique used to improve the appearance of acne scarring.

**Objective:**

To comprehensively review the medical literature regarding skin needling as a treatment for acne scarring.

**Methods:**

A literature search was performed using the PubMed, Medline, and Embase databases, in addition to reviewing the bibliographies of relevant articles.

**Results:**

Ten studies presented patients treated with skin needling alone, while eight studies discussed skin needling in combination with other treatments for acne scarring. All studies showed improvements in scarring after needling, with 12 reporting statistical significance. The median number of treatments when needling was used alone was three, the median duration between treatments was 4 weeks, and the median needle length used was 1.5 mm. Reported adverse events were infrequent and included post-inflammatory hyperpigmentation, “tram track” scarring, acne, and milia. There were no reports of bacterial infections.

**Limitations:**

The studies reviewed were heterogeneous in design and of variable validity, with some not reporting statistical significance.

**Conclusion:**

There is moderate evidence to suggest that skin needling is beneficial and safe for the treatment of acne scarring. However, double-blinded, randomized controlled trials are required to make more definitive conclusions.

## Introduction

**Fig. 1:**
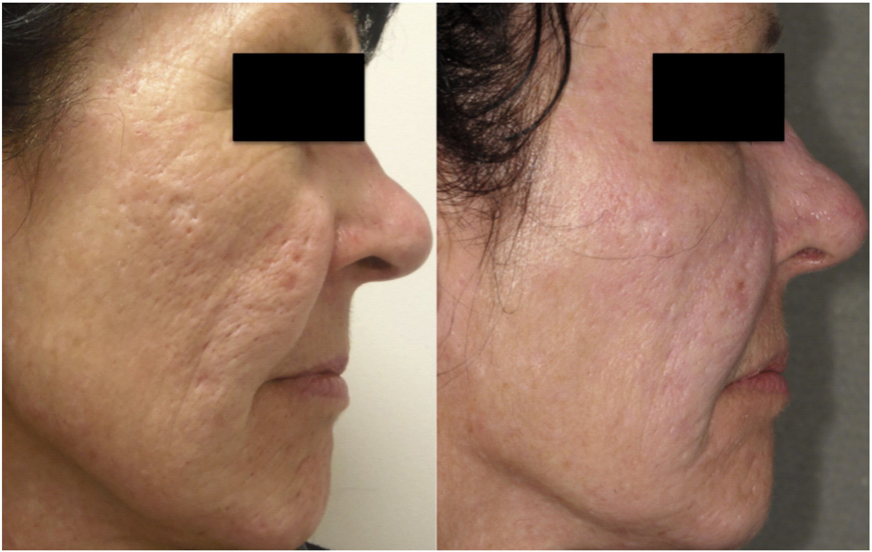
An improvement in acne scarring and photodamage in a patient treated with one session of skin needling.

Skin needling is a technique predominantly used to improve the appearance of cutaneous scarring and photodamage (Fig. 1). Fine needles puncture the skin, resulting in increased dermal elastin and collagen, collagen remodeling, and thickening of the epidermis and dermis (Aust et al., 2008a, Aust et al., 2010a, Aust et al., 2010b, Aust et al., 2011, Fabbrocini et al., 2011a, Fernandes, 2005, Fernandes and Signorini, 2008, Kim et al., 2011, Park et al., 2012, Schwarz and Laaff, 2011). Additionally, skin needling creates small channels, which increase the absorption of topically applied preparations (Badran et al., 2009, Kalluri et al., 2011), a property which has been used in various dermatological treatments (Bencini et al., 2012, Budamakuntla et al., 2013, Clementoni et al., 2010, Fabbrocini et al., 2011b, Fabbrocini et al., 2014a, Kang et al., 2008, Torezan et al., 2013, Yoon et al., 2008, Yoon et al., 2010).

Skin needling for cutaneous scarring was introduced into the medical literature in 1997, when tattooing without pigment was used to abrade facial scars, improving their quality and color (Camirand and Doucet, 1997). In 1998, Desmond Fernandes, a plastic surgeon from South Africa, designed a hand-held device composed of a rolling barrel with multiple protruding needles and used it for a technique he termed “percutaneous collagen induction.” He later published his experience using this technique for various dermatological conditions, including acne scarring (Fernandes, 2005, Fernandes and Signorini, 2008).

Both manual and electronic hand-held skin needling devices are now widely available as low-cost therapies for the treatment of acne scarring (Doddaballapur, 2009, Dogra et al., 2014), yet, to our knowledge, there has been no dedicated review of the medical literature regarding this topic.

## Objective

The objective of this study was to comprehensively review the medical literature regarding skin needling as a treatment for acne scarring.

## Material and methods

A literature search was performed using the PubMed, Medline 1946-to-present, and Embase classic plus Embase 1947-to-present databases. Search terms included ‘skin needling’, ‘microneedling’, ‘needle dermabrasion’, ‘tattooing without pigment’, ‘dry tattooing’, ‘percutaneous collagen induction therapy’, and ‘dermaroller’. All terms are used in the medical literature to describe skin needling and were combined with the term acne. The search was performed without limitations or language restrictions. All bibliographies within the relevant articles were reviewed for their relevance.

## Results

Nineteen articles included patients with acne scarring treated with skin needling. Included were nine prospective observational studies (Beretta et al., 2008, Dogra et al., 2014, Fabbrocini et al., 2009, Fabbrocini et al., 2014b, Garg and Baveja, 2014, Kang et al., 2009, Kim, 2008, Majid, 2009, Schwarz and Laaff, 2011), nine prospective controlled studies (Alam et al., 2014, Fabbrocini et al., 2011c, Gadkari and Nayak, 2014; Kofal et al., 2014; Leheta et al., 2011, Leheta et al., 2014a, Leheta et al., 2014b, Mohammed, 2013, Sharad, 2011), and one case report (Pahwa et al., 2012). Additionally, one prospective observational study likely contained patients with acne scarring (Aust et al., 2008a), but the method was unclear. Eighteen articles were in English and one was in Italian, which was translated. All studies were critically evaluated and are summarized in tables that can be found in the Table I, Table II, Table III.

### Efficacy

The majority of the studies reviewed included an objective scar scoring system to measure efficacy, most commonly the grading systems designed by Goodman and Baron (Goodman and Baron, 2006a, Goodman and Baron, 2006b). Others used subjective scar scoring scales, subjective improvement scales, and patient satisfaction scales. For the purpose of this review, if an objective scar scoring scale was not used, the results of a subjective scale were used to evaluate efficacy.

### Studies evaluating the efficacy of skin needling alone as a treatment for acne scarring

Six studies measured the efficacy of skin needling alone as a treatment for acne scarring (Alam et al., 2014, Beretta et al., 2008, Camirand and Doucet, 1997, Fabbrocini et al., 2009, Fabbrocini et al., 2014b, Majid, 2009). All studies showed improvements in scar severity scores compared to baseline, with statistical significance reported in all except one. One study was a randomized placebo controlled trial (Alam et al., 2014); the remaining five were prospective observational trials, which are subject to a high risk of selection bias and inherently lower validity.

The first study published included 20 patients assessed before and after treatment by two physicians, blinded to pretreatment scores using the Goodman and Baron grading systems (Beretta et al., 2008). After treatment, there was a statistically significant improvement in scores, but it was unclear how this was calculated.

A similar study included 37 patients with facial scarring, 32 of whom had acne scarring (Majid, 2009). A single dermatologist consecutively graded patients using the qualitative Goodman and Baron grading system. Twenty-seven of the 31 patients with acne scarring who completed the study had improved scar grades, but there was no statistical analysis of the results.

An Italian group published two further observational studies (Fabbrocini et al., 2009, Fabbrocini et al., 2014b). The first (2009) included 32 patients consecutively graded by the same dermatologist using the qualitative Goodman and Baron grading system. After treatment, there was a statistically significant reduction in the mean severity grading. A second, larger study (2014b) included 60 patients whose scar severity was evaluated from photographs using the Global Aesthetic Improvement Scale. After treatment, there was also a statistically significant reduction in severity grading.

A recent uncontrolled study form India (Dogra et al., 2014) included 36 patients assessed with the acne scar assessment tool described by Peterson et al. (2011). After treatment, there was a statistically significant improvement in the mean scar grading from 11.73 to 6.5, although six patients did not complete the study, five because of treatment-related complications including severe post-inflammatory hyperpigmentation and tram-trek scarring.

The most rigorous study was a placebo-controlled, split-face trial from the United States, which included 20 participants randomized to receive either skin needling with topical anesthetic or topical anesthetic alone to either side of the face (Alam et al., 2014). Two dermatologists blinded to the intervention graded standardized photographs with the quantitative Goodman and Baron grading system. At 6 months posttreatment, there was a statistically significant decrease in the mean grade of the skin-needled side compared to the placebo side, with a mean difference in scores of 3.4 compared to 0.4. Five patients dropped out before the study protocol was initiated. There was no blinding of the patients.

An additional study included a prospective sub-analysis of 15 patients with “scars and stretch marks,” but it was not clear if the group contained patients with acne scarring (Aust et al., 2008a). Patients were a part of a larger group included in a retrospective analysis of skin needling used for a variety of conditions including acne scarring. The subgroup demonstrated a statistically significant improvement based on the Vancouver Scar Scale (Baryza, 1995) and Observer Scar Assessment Scale as assessed by two independent observers.

### Studies evaluating the efficacy of skin needling compared to other methods of treatment for acne scarring

A total of three studies compared skin needling to other methods of treatment for acne scarring.

An Egyptian group (Leheta et al., 2011) randomized 30 participants to receive either skin needling or the focal application of 100% trichloroacetic acid (TCA) using the CROSS (chemical reconstruction of skin scars) method (Lee et al., 2002). A blinded dermatologist scored a 68% mean improvement in the needling group and a 75% improvement in the TCA group, with no statistically significant difference between groups. Participants were not blinded and three participants in the TCA group dropped out and were not included in the analysis on an intention-to-treat basis.

An Italian group compared skin needling to skin needling combined with the topical application of platelet-rich plasma (PRP) in a split-face trial of 12 patients (Fabbrocini et al., 2011c). After treatment, all scores were reduced but the PRP group had an overall mean lower severity score. The statistical significance of this result was not reported. There was also no mention of blinding, either of the investigator or the patient, or randomization to which side received each treatment.

A study from India sequentially enrolled 30 patients and compared skin needling to the combination of skin needling alternating with 35% glycolic acid (GA) peels (Sharad, 2011). Scars were graded by the treating dermatologist using the Echelle d’Evaluation clinique des Cicatrices d’acne classification (Dreno et al., 2007). The combination treatment resulted in a statistically significant greater mean improvement of 63% compared to 31%. The patients and the assessors were not blinded.

### Studies evaluating the efficacy of skin needling in conjunction with other treatments for acne scarring

Skin needling was used in eight studies to increase the penetration of topically applied preparations and alongside other treatments to synergistically improve efficacy.

An Egyptian group randomly assigned 24 patients to receive skin needling and 20% TCA or deep skin peeling using 60% phenol (Leheta et al., 2014a). Live assessment by a dermatologist blinded to the intervention gave statistically significant mean improvements in scores of 70% in the combination group and 75% in the deep phenol peel group with no statistically significant difference between two. A second study by the group randomly assigned 39 patients to skin needling and 20% TCA (group I), fractional thermolysis (group II), or a combination of both treatments (group III) (Leheta et al., 2014b). The same blinded dermatologist scored patients and gave an improvement in mean severity scores of 60%, 62%, and 78%, respectively. There was no statistically significant difference between groups I and II, but there was between groups I and II versus group III. One patient from group I was lost to follow-up and was included in the analysis on an intention-to-treat basis. Two patients from each group in the first study dropped out and were not included in the analysis. In both studies the patients were not blinded.

Another study combined skin needling, subcision, and 15% TCA in 50 patients (Garg and Baveja, 2014). Photographs were graded by the same nontreating physician using the qualitative Goodman and Baron grading system. Scar grades improved in all patients and although the process of statistical analysis was mentioned, it did not appear to be performed and presented.

A Korean study (Kang et al., 2009) assessed the combination of the focal application of 100% TCA, skin needling using a 29-gauge needle, subcision (Orentreich and Orentreich, 1995), and fractional thermolysis in 35 patients. An independent physician scored patients using the acne severity scale described by Lipper and Perez (2006). Ten patients completed the study, with scores improving in all patients. Statistical significance of the results was not reported and there was a large loss to follow-up.

An Indian study compared skin needling combined with subcision to subcision combined with cryorolling (Gadkari and Nayak, 2014). Cryorolling consisted of dipping the needling device into liquid nitrogen immediately before the procedure. Thirty-seven patients were randomized to have both procedures to either side of their face and a blinded observer scored standardized photographs using the qualitative and quantitative Goodman and Baron grading systems. Both treatments resulted in statistically significant improvements in mean quantitative gradings of 57% in the cryoroller group and 40% in the needling group. The difference between the two was statistically significant. Seven patients dropped out of the study and it was unclear which group they were from and whether they were included with intention to treat.

Another Egyptian study compared 45 patients equally randomized into three groups to receive either skin needling combined with the topical application of PRP, the focal application of 100% TCA, or intralesional dermal injections of PRP (Nofal et al., 2014). Photographs were assessed by two blinded dermatologists using the qualitative Goodman and Baron grading system. All three treatments resulted in statistically significant improvements in scar grades with no difference between them.

Finally, two separate studies looked at the combination CO_2_ laser and skin needling using a 26-gauge needle. The first included 35 patients who all had improvements in a 4-point improvement scale. There was no statistical analysis of the results (Kim, 2008). The second randomized 60 patients to compare laser and skin needling to laser alone (Mohammed, 2013). Patients were assessed by three independent observers blinded to the treatment using the quantitative Goodman and Baron grading system. After treatment there was a statistically significant improvement in scores in both groups with no statistically significant difference between them.

### Histological changes

One study published histological sections before and after skin needling treatment of 10 patients with posttraumatic and acne scarring (Schwarz and Laaff, 2011). A blinded dermatologist and pathologist concluded that, in seven patients, there was a noticeable increase in elastin correlating with the depth of needle penetration. Increases in collagen and dermal thickness, but no change in epidermal thickness, were also noted. These results are similar to studies treating patients for other scarring conditions. An increase in collagen and elastin was seen in two studies treating patients with striae distensae (Aust et al., 2010b, Park et al., 2012), with one additionally showing an increase in epidermal thickness (Park et al., 2012). A study treating burn scars showed an increase in collagen (Aust et al., 2010a). Increases in collagen, elastin, and epidermal and dermal thickness have also been seen with skin needling in nonscarring conditions such as photodamage and skin laxity (Aust et al., 2008a, Aust et al., 2011, Fabbrocini et al., 2011a, Fernandes, 2005, Fernandes and Signorini, 2008, Kim et al., 2011).

### Optimal number of treatments, duration between treatments, and needle length

There have been no studies directly evaluating the optimal number of treatments, time between treatments, or needle length. The median number of treatments in studies using skin needling alone was three (Alam et al., 2014, Beretta et al., 2008, Dogra et al., 2014, Fabbrocini et al., 2009, Fabbrocini et al., 2011c, Fabbrocini et al., 2014b, Leheta et al., 2011, Majid, 2009, Schwarz and Laaff, 2011, Sharad, 2011), with a range of one to five treatments. The median duration between treatments was 4 weeks, with a range of 2 to 8 weeks. The median needle length used was 1.5 mm, ranging from 1 mm to 3 mm. No studies compared needle thickness or degree of pressure applied.

### Adverse events

**Fig. 2:**
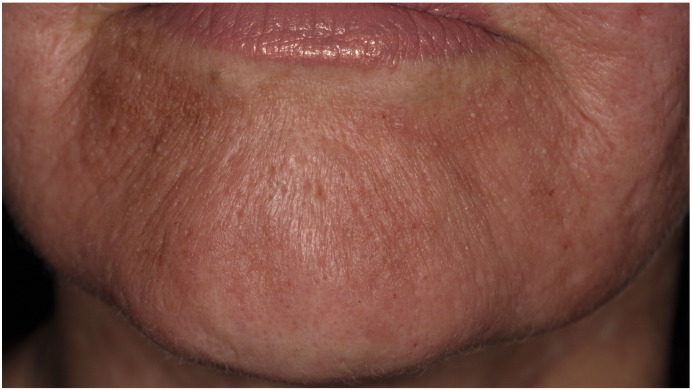
Development of erythema, crusting, and pustules in a patient one day after skin needling.

Eighteen patients out of 246, over 10 studies (Alam et al., 2014, Beretta et al., 2008, Dogra et al., 2014, Fabbrocini et al., 2009, Fabbrocini et al., 2011c, Fabbrocini et al., 2014b, Leheta et al., 2011, Majid, 2009, Schwarz and Laaff, 2011, Sharad, 2011) treating patients with skin needling alone, had adverse events reported. Skin needling is expected to cause temporary erythema, pain, a burning sensation, edema, bleeding, or a serous ooze resolving with crusting or scabbing (Alam et al., 2014, Beretta et al., 2008, Dogra et al., 2014, Fabbrocini et al., 2009, Fabbrocini et al., 2011c, Fabbrocini et al., 2014b, Leheta et al., 2011, Majid, 2009, Sharad, 2011). Bruising and hematomas are also expected, particularly over bony prominences (Dogra et al., 2014, Fabbrocini et al., 2014b, Schwarz and Laaff, 2011), The development of “tram trek” scarring was reported with 2-mm needles in one case report (Pahwa et al., 2012). This phenomenon also occurred in a study using 1.5-mm needles (Dogra et al., 2014), but not in a study using 2.5-mm needles (Gadkari and Nayak, 2014). It is therefore unclear if this phenomenon is related to needle length. There were no other reports of scarring. Other adverse events included the development of acne and the formation of milia (Beretta et al., 2008, Leheta et al., 2011, Sharad, 2011) (see Fig. 2). Serious adverse events worth noting when skin needling was used for other dermatological conditions included facial allergic granuloma and systemic hypersensitivity reactions, possibly related to topical products put on the skin before needling or to the needles themselves (Patsou, 2013, Soltani-Arabshahi et al., 2014).

### Infection as an adverse event

No studies reported bacterial infections after treatment, although some opted for topical or oral antibiotic prophylaxis (Alam et al., 2014, Gadkari and Nayak, 2014, Majid, 2009, Sharad, 2011). One study reported infections with herpes simplex virus (HSV), but it was unclear if these patients had acne scarring (Aust et al., 2008a). Reports of HSV infections have been noted in other articles (Fernandes, 2005, Torezan et al., 2013) and oral acyclovir was given to patients with a history of HSV in at least one study including patients with acne scarring (Alam et al., 2014).

### Post-inflammatory hyperpigmentation as an adverse event

In an early study using rats, post-inflammatory hyperpigmentation after skin needling was found to be unlikely (Aust et al., 2008b). Of the patients treated with skin needling alone, nine developed postinflammatory hyperpigmentation (Dogra et al., 2014, Majid, 2009, Sharad, 2011); all had skin phototypes of three or greater except one, where the skin phototype was not mentioned. Not all studies reported skin types, but in at least four studies (Alam et al., 2014, Dogra et al., 2014, Fabbrocini et al., 2014b, Sharad, 2011), there were a total of 105 patients with skin phototypes of three or greater.

## Conclusion

Skin needling is a relatively simple, cost-effective technique used for the treatment of acne scarring. A review of the current literature suggests that it has moderate efficacy. However, the evidence for this is limited as it is based on predominantly observational studies, which are heterogeneous in design with some lacking statistical analysis and internal validity. Skin needling was shown to work well in combination with other treatments for acne scarring, but the results of each study were specific for each treatment and lacked external validity. Skin needling appeared to be safe with a low frequency of side effects. No consensus has been reached on the use of antibiotic prophylaxis. Double-blinded randomized controlled trials are needed to further evaluate efficacy, and specific studies are needed to define the optimal number of treatments, duration between each treatment, and needle depth, and to further characterize adverse reactions.

The following are the supplementary data related to this article.

Table I Studies Evaluating the Efficacy of Skin Needling Alone as a Treatment for Acne Scarring.

Table II Studies Evaluating the Efficacy of Skin Needling Compared to Other Methods of Treatment for Acne Scarring.

Table III Studies Evaluating the Efficacy of Skin Needling in Conjunction with Other Treatments for Acne Scarring.
