# Supplementary material for: Skin needling as a treatment for acne scarring: An up-to-date review of the literature
Source: Int J Womens Dermatol. 2015 Apr 10;1(2):77–81. doi: 10.1016/j.ijwd.2015.03.004 (PMC5418754; doi:10.1016/j.ijwd.2015.03.004)
Supplement: Table I — Studies Evaluating the Efficacy of Skin Needling Alone as a Treatment for Acne Scarring. [file mmc1.doc]

| **Author, country** | **Journal (Year)** | **Study design** | **Number of patients with acne scarring** | **Blinding** | **Randomization** | **Primary measure of efficacy** | **Number of treatments** | **Duration between treatments (weeks)** | **Timing of the final assessment of efficacy** | **Losses to follow up** | **Statistical significance of the results measuring efficacy** | **Adverse events** | **Needle length used (mm)** | **Skin phototype** |
| --- | --- | --- | --- | --- | --- | --- | --- | --- | --- | --- | --- | --- | --- | --- |
| Beretta et al., Italy | *Journal of Plastic Dermatology* (2008) | Prospective observational, uncontrolled | 20 (51 in the study in total) | Assessor blinded to pretreatment scores | N/A | Goodman and Baron grading systems | 1 | N/A | 6 months after the final treatment | Not reported | Yes | 2 developed acne. 2 developed milia | Not reported | Not reported |
| Majid, India | *Journal of Cutaneous and Aesthetic Surgery* (2009) | Prospective observational, uncontrolled | 32 (37 in the study in total) | Not reported | N/A | Qualitative Goodman and Baron grading system | 3 or 4 | 4 | 2 months after the final treatment | 1 patient with acne scarring | Not reported | 1 developed post-inflammatory hyperpigmentation | 1.5 | Not reported |
| Fabbrocini et al., Italy | *Clinical and Experimental Dermatology* (2009) | Prospective observational, uncontrolled | 32 | Not reported | N/A | Qualitative Goodman and Baron grading system | 2 | 8 | 8 weeks after the final treatment | 0 | Yes | - | 1.5 | Not reported |
| Fabbrocini et al., Italy and the United Kingdom | *Journal of Dermatological Treatment* (2014b) | Prospective observational, uncontrolled | 60 | Not reported | N/A | Global Aesthetic Improvement Scale | 3 | 4 | 13 months after the first treatment | 0 | Yes | - | 1.5 | Type 1, 2: 10   Type 3, 4, 5: 45   Type 6: 5 |
| Dogra et al., India | *Journal of Cosmetic Dermatology* (2014) | Prospective observational, uncontrolled | 36 | Not reported | N/A | Acne scar assessment tool described by Peterson et al. | 5 | 4 | 1 month after the final treatment | 6 | Yes | 5 developed post-inflammatory hyperpigmentation. 2 developed tram-trek scarring | 1.5 | Type 4:14  Type 5:16 |
| Alam et al., United States | *Journal of the American Medical Association Dermatology* (2014) | Prospective placebo, controlled | 20 | Assessor blinded to intervention. Patients not blinded | Randomization of skin needling or placebo to either side of the face | Quantitative Goodman and Baron grading system | 3 | 2 | 3 and 6 months after the first treatment | 5 | Yes | - | 1 or 2 | Type 1:1  Type 2:4  Type 3:6  Type 4:3  Type 5:1 |
| Aust et al., Germany, South Africa and the United States | *Plastic Reconstructive Surgery* (2008a) | Prospective observational, uncontrolled | Unclear | Not reported | N/A | Vancouver Scar Scale, Observer Scar Assessment Scale | 1 to 4 | Not reported | 12 months after first treatment | Not reported | Yes | 2 developed HSV infections but it was not clear if they had acne scarring or not | 1 to 3 | Not reported |

**Table I: Studies Evaluating the Efficacy of Skin Needling Alone as a Treatment for Acne Scarring**

N/A, not applicable; HSV, herpes simplex virus.
