# Supplementary material for: Skin needling as a treatment for acne scarring: An up-to-date review of the literature
Source: Int J Womens Dermatol. 2015 Apr 10;1(2):77–81. doi: 10.1016/j.ijwd.2015.03.004 (PMC5418754; doi:10.1016/j.ijwd.2015.03.004)
Supplement: Table II — Studies Evaluating the Efficacy of Skin Needling Compared to Other Methods of Treatment for Acne Scarring. [file mmc2.doc]

| **Author, country** | **Journal (Year)** | **Study design** | **Number of patients with acne scarring** | **Blinding** | **Randomization** | **Primary measure of efficacy** | **Number of treatments** | **Duration between treatments (weeks)** | **Timing of final assessment of efficacy** | **Losses to follow up** | **Statistical significance of results measuring efficacy** | **Adverse events** | **Needle length used (mm)** | **Skin phototype** |
| --- | --- | --- | --- | --- | --- | --- | --- | --- | --- | --- | --- | --- | --- | --- |
| Leheta et al., Egypt | *Dermatologic Surgery* (2011) | Prospective, controlled | 30 (15 treated with skin needling and 15 with 100% TCA) | Assessor blinded. Patients not blinded | Randomization of either treatment to each side of the face | Severity score of 1-3 for each scar then each score combined to get a total score | 4 | 4 | 4 weeks after the final treatment | 3 | Yes | 2 developed acne | 1.5 | Type 2:1  Type 3:15  Type 4:11  (not broken down in terms of treatment) |
| Fabbrocini et al., Italy | *Cosmetic Dermatology* (2011c) | Prospective, controlled | 12 (6 treated with skin needling and 6 with skin needling and topical PRP) | Not reported | No | 0-10 scar severity score | 2 | 8 | 32 weeks after the last treatment | 0 | Not reported | - | 1.5 | Not reported |
| Sharad., India | *Journal of Cosmetic Dermatology* (2011) | Prospective, controlled | 30 (15 treated with skin needling and 15 with skin needling and GA peel) | Not reported | No | Echelle d’Evaluation clinique des Cicatrices d’acne classification | 5 treatments each | 6 | 3 months after the last treatment | 0 | Yes | 1 developed milia and 3 developed post-inflammatory hyperpigmentation in the skin-needling group. 1 patient developed milia in the combined group | 1.5 | Type 3-4:15 (in the group receiving skin needling) |

**Table II: Studies Evaluating the Efficacy of Skin Needling Compared to Other Methods of Treatment for Acne Scarring**

TCA, trichloroacetic acid; PRP, platelet-rich plasma; GA, glycolic acid.
