# Supplementary material for: Skin needling as a treatment for acne scarring: An up-to-date review of the literature
Source: Int J Womens Dermatol. 2015 Apr 10;1(2):77–81. doi: 10.1016/j.ijwd.2015.03.004 (PMC5418754; doi:10.1016/j.ijwd.2015.03.004)
Supplement: Table III — Studies Evaluating the Efficacy of Skin Needling in Conjunction with Other Treatments for Acne Scarring. [file mmc3.doc]

**Table III: Studies Evaluating the Efficacy of Skin Needling in Conjunction with Other Treatments for Acne Scarring**

| **Author, country** | **Journal (Year)** | **Study design** | **Number of patients with acne scarring** | **Blinding** | **Randomization** | **Primary measure of efficacy** | **Number of treatments** | **Duration between treatments (weeks)** | **Timing of final assessment of efficacy** | **Losses to follow up** | **Statistical significance of results measuring efficacy** | **Adverse events** | **Needle length used (mm)** |
| --- | --- | --- | --- | --- | --- | --- | --- | --- | --- | --- | --- | --- | --- |
| Leheta et al., Egypt | *Journal of Dermatological Treatment* (2014a) | Prospective, controlled | 24 (12 skin needling and TCA,  12 deep phenol peel) | Assessor blinded. Patients not blinded | Randomization to either treatment | Severity score of 1-3 for each scar then each score combined to get a total score | 4 | 6 | 8 months after the first treatment | 4 total (2 in each group) | Yes | - | 1.5 |
| Leheta et al., Egypt | *Journal of Dermatological Treatment* (2014b) | Prospective, controlled | 29 (13 in group I: 20% TCA, 13 in group II: fractional thermolysis, 13 in group III: a combination of both) | Assessor blinded. Patients not blinded | Randomization to either treatment | Severity score of 1-3 for each scar then each score combined to get a total score | Group 1 and 2: 6  Group 3: 3 of each treatment | 4 | 12 months after the first treatment | 1 in skin needling and TCA group included in the analysis | Yes | - | 1.5 |
| Garg and Baveja, India | *Journal of Cutaneous and Aesthetic Surgery* (2014) | Prospective observational, uncontrolled | 50 (skin needling, subcision, and 15% TCA) | Unclear: “nontreating physician” | N/A | Qualitative Goodman and Baron grading system | 6 of each treatment | 2 | 1 month after final treatment | 1 | Not reported | 2 developed acne | 1.5 |
| Kang et al., Korea | *Journal of Cosmetic and Laser Therapy* (2009) | Prospective observational, uncontrolled | 35 (100% TCA, skin needling, subcision, and fractional thermolysis) | Unclear: “independent evaluator” | N/A | Acne severity scale described by Lipper and Perez | 2 treatments of 100% TCA, skin needling, and subcision. 3-4 treatments of fractional thermolysis | 8 to 12 weeks for 100% TCA, skin needling and subcision. Every 3-4 weeks for fractional thermolysis | 3 months after the final treatment | 25 | Not reported | - | 29-gauge needle. Depth unclear |
| Gadkari and Nayak, India | *Journal of Cosmetic Dermatology* (2014) | Prospective, controlled | 37 (skin needling combined with subcision and subcision combined with cryorolling to each side of the face) | Assessor blinded. Patients not blinded | Randomization of either treatment to each side of the face | Quantitative Goodman and Baron grading system | 3 | 1 | 6 months after the first treatment | 7 (unclear from which group) | Yes | 5 in cryoroller group developed post-inflammatory hyperpigmentation | 2.5 |
| Nofal et al., Egypt | *Dermatologic Surgery* (2014) | Prospective, controlled | 45 (15 skin needling combined with PRP, 15 focal application of 100% TCA, 15 intralesional dermal injections of PRP) | Assessor blinded. Patients not blinded | Randomization to either treatment | Qualitative Goodman and Baron grading system | 3 | 2 | 2 weeks after final treatment | 0 | Yes | - | 2 |
| Kim, Korea | *Journal of Cosmetic and Laser Therapy* (2008) | Prospective observational, uncontrolled | 35 (CO_2_ laser and skin needling) | No reported | N/A | Physician 4-point improvement scale | 5 | 2 to 3 | 3 months after the final treatment | 0 | Not reported | - | 26-gauge needle to 1 mm |
| Mohammed, Egypt | *Journal of Cosmetic and Laser Therapy* (2013) | Prospective, controlled | 60 (30 laser and skin needling, 30 laser alone) | Assessor blinded. Patients not blinded | Randomization to either treatment | Quantitative Goodman and Baron grading system | 4 | 3 | 3 months after the final treatment | 0 | Yes | - | 26-gauge needle to 1 mm |

TCA, trichloroacetic acid; N/A, not applicable; PRP, platelet-rich plasma.
